# Ticks (Acari: Ixodidae) and tick-borne diseases affecting communal cattle and the control methods practiced by farmers in the Eastern Cape Province of South Africa

**DOI:** 10.14202/vetworld.2025.746-754

**Published:** 2025-03-31

**Authors:** N. Nyangiwe, S. Matthee

**Affiliations:** 1 Department of Agriculture and Animal Health, University of South Africa, Florida, 1710, South Africa; 2 Department of Agriculture, Döhne Agricultural Development Institute, Private Bag X15, Sutterheim 4930, South Africa; 3 Department of Conservation Ecology and Entomology, University of Stellenbosch, Stellenbosch, 6201, South Africa

**Keywords:** acaricide resistance, climate change, communal cattle, South Africa, tick-borne diseases, ticks

## Abstract

**Background and Aim:**

Ticks and tick-borne diseases (TBDs) pose significant threats to cattle farming, impacting livestock health, productivity, and economic sustainability. In communal farming systems, the challenges of tick control are exacerbated by limited resources, acaricide resistance, and climate change. This study assesses communal cattle farmers’ knowledge, attitudes, and practices regarding ticks, TBDs, and the control measures implemented in the Eastern Cape Province (ECP) of South Africa.

**Materials and Methods:**

A cross-sectional survey was conducted using structured questionnaires administered to 100 cattle farmers across 20 communities in four vegetation types: Albany coastal belt (ACB), Amathole montane grassland (AMG), Bhisho thornveld (BT), and Great fish thicket (GFT). Data were analyzed using descriptive statistics, Pearson’s Chi-square tests, and Kruskal-Wallis tests to determine associations between farmer demographics, livestock management practices, and the prevalence of TBDs.

**Results:**

Most respondents (85%) were male, with an average age of 60 years, and 65% had only primary education. Livestock ownership varied across vegetation types, with cattle numbers ranging between 12.8 ± 1.17 and 15.6 ± 1.35 per farmer. Tick infestation was perceived as a major constraint, with adult cattle more affected than calves (χ^2^ = 15.98, p < 0.001). The most commonly reported TBDs were redwater (100%), gallsickness (90%), and heartwater (43%), with heartwater absent in AMG. Tick control methods included plunge dipping (90%) and the use of alternative treatments such as used motor oil (54%) and Jeyes fluid (35%). Acaricide inefficacy, poor mixing practices, and the uncontrolled movement of cattle were identified as major constraints to effective tick management.

**Conclusion:**

Communal cattle farmers in the ECP recognize ticks and TBDs as critical challenges, with variations in TBD prevalence linked to vegetation type. Ineffective acaricide use and resistance are growing concerns, necessitating improved extension services and farmer education. Sustainable tick management strategies should integrate scientific knowledge with indigenous practices to enhance livestock health and productivity in communal farming systems.

## INTRODUCTION

Worldwide ticks and tick-borne diseases (TBDs) negatively affect the productivity, condition, fertility, and health of animals in the cattle farming industry [1, 2]. The negative impact of ticks on cattle production is both directly (by heavy infestations and skin damage) and indirect, through the transmission of tick-borne pathogens, which can affect growth rates and health and lead to herd losses [3]. The impact of TBDs on cattle farming and animal production, in general, is particularly extensive in tropical and subtropical regions [4, 5]. De Castro [4] estimated the global costs of TBDs for cattle to be more than US$ 15 billion per annum. A more recent and realistic estimate of the impact of ticks and TBDs is in the vicinity of US$ 22–30 billion per annum [6]. Regarding African countries, estimates of loss due to TBDs are equally high, with countries such as Tanzania recording US$364 million [7] and South Africa about ZAR 1.059 billion per annum in 2023 [8]. Communal farmers face different challenges in terms of ticks and TBDs, such as a lack of dipping material because government dipping subsidies are not maintained throughout the year. This leads poor resource farmers to have animals with heavy tick loads, especially during the hot-wet season.

The Eastern Cape Province (ECP) has the highest number of cattle (approximately 3.2 million) in South Africa, followed by KwaZulu Natal Province (2.7 million) and Free State Province (2.3 million) [9]. TBD poses a major threat to most small-scale cattle farmers on communal rangelands in the ECP [10, 11]. The significance of TBDs in the province is evident in the fact that there are more than a thousand communal dip tanks in the province and the cost associated with plunge dipping, which amounts to R 98,000 (approximately 5,192, 49 USD) in the communal sector of the ECP. The most common tick-related diseases prevalent in the ECP include heartwater (caused by *Ehrlichia ruminantium*), which is transmitted by *Amblyomma* *hebraeum*; bovine babesiosis (caused by *Babesia bigemina* and *Babesia bovis*), and anaplasmosis (caused by *Anaplasma marginale*), which are transmitted by *Rhipicephalus decoloratus* and *R. microplus* [12–15].

The ECP of South Africa is characterized by almost all-year rainfall (500 mm–900 mm in the cool dry and hot wet seasons) and mild temperatures (5°C–35°C). Ninety percentages of land in the ECP is rangeland, which is more suitable for livestock farming than crop production [16]. The major biomes in the province include grassland and the Albany Thicket, with several types of vegetation occurring across altitudinal gradients. Specifically, Bhisho thornveld (BT) (468 m above mean sea level) (AMSL) and Amathole montane grassland (AMG) (848 m AMSL) were found at higher elevations than Great Fish Thicket (GFT) (193 m AMSL) and Albany coastal belt (ACB) (98 m AMSL). The type of vegetation can influence the distribution of tick species by either influencing host diversity and distribution or providing different microclimatic conditions that can affect the survival of tick species [17, 18]. However, the type of TBD will also be affected by vegetation type, which can influence the knowledge and perceptions of life stock owners.

Despite the recognized economic and health burdens posed by ticks and TBDs in communal cattle farming systems, there remains limited empirical data on the interplay between vegetation types, farmer knowledge, and the prevalence of TBDs in the ECP of South Africa. In addition, the effectiveness of current tick control strategies and farmer adaptation measures to acaricide resistance and climate change remains insufficiently explored. The need for region-specific sustainable tick management practices that integrate both scientific and indigenous knowledge is yet to be adequately addressed.

This study aims to assess communal cattle farmers’ knowledge, attitudes, and practices regarding ticks and TBDs and to evaluate the influence of vegetation types on the prevalence of TBDs in the ECP of South Africa. Furthermore, the study seeks to identify key constraints in tick control measures and propose sustainable management strategies to enhance livestock health and productivity.

## MATERIALS AND METHODS

### Ethical approval and Informed consent

This study was approved by the Ethics Committee Focusing on Humans at Stellenbosch University (reference no: DESC_Nyangiwe2012). No animals were used in this study. All respondents were informed about the purpose of the study, and their participation was voluntary. The farmers were guaranteed the privacy of the information provided during the interviews.

### Study period and location

The study was conducted October 2022 to February 2023 at communal farming areas in the Amathole District Municipality of the ECP.

**Figure 1:**
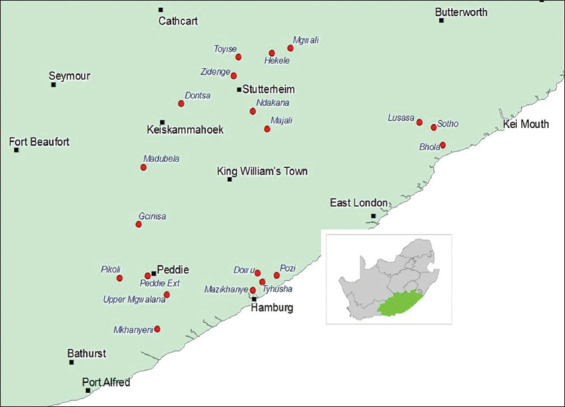
(a) Geographic position of the Eastern Cape Province in South Africa and (b) communities that participated (dots) in the Amathole District Municipality of the Eastern Cape Province in South Africa [Source: The map was generated using QGIS v 2.6.1].

The ECP is located on the eastern coast of South Africa. The province covers an area of 168 966 km^2^, which is 13.5% of South Africa’s total land area. It has a population of more than 6.5 million people and is the second-largest province in South Africa [9]. Twenty cattle-farming communities were randomly selected within four vegetation types; namely, ACB, AMG, BT, and GFT [19] (Figure 1).

The vegetation of the Albany Thicket Biome is described in general as a dense, woody, semi-succulent, and thorny vegetation type with an average height of 2–3 m. Albany Thicket Biome is found in semi-arid Eastern and Western Cape areas with annual rainfall of 200–950 mm. Thicket vegetation growing close to the coast experiences less extreme climatic variability due to the influence of the ocean.

The ACB occurs at 10–400 m above sea level. The mean monthly maximum and minimum temperatures are 32°C and 5°C, respectively. Graminoids include *Cynodon dactylon*, *Dactyloctenium australe*, *Digitaria natalensis*, *Ehrharta calycina*, *Eragrostis capensis*, *Eragrostis curvula*, *Eragrostis plana*, *Heteropogon contortus*, *Panicum deustum*, *Panicum maximum*, *Setaria sphacelata*, *Sporobolus africanus*, *Themeda triandra*, *Tristachya leucothrix*, and *Cymbopogon marginatus*.

The vegetation under AMG is characterized by medium-height grasslands interspersed with *Acacia karroo* woodlands. The dominant grass species are *Eragrostis chloromelas*, *E. curvula*, *S. africanus*, *H. contortus*, *T. leucothrix* and *T. triandra*There are subspecies of low shrubs, such as *Anthospermum rigidum* and *Felicia filifolia*, that are present. The altitudes range between 650 and 1500 m above sea level and have annual rainfall between 500 and 700 mm while the maximum and minimum temperatures are 25°C and 2°C, respectively. Winter frost is not common in the south eastern part of this vegetation unit, but it is more common (up to 80 days/year) in the western and north western regions [19].

The BT occurs at an altitude of 500 m above sea level and has an average annual rainfall of 480 mm; the maximum and minimum temperatures are 37°C and 13°C, respectively. The grass species included *Digitaria eriantha*, *Aristida congesta*, *C. dactylon*, *Eragrostis* spp., *Sporobolus fimbriatus*, *T. triandra*, and *S. africanus*. *Maytenus polyacantha*, *Scutia myrtina*, and *A. karroo* are the most dominant tree species [19].

GFT is found at altitudes of about 700–1,300 m and has an average annual rainfall between 500 mm and 840 mm. The mean monthly maximum and minimum temperatures are 38.6°C and −1.0°C, respectively.

### Data collection

Face-to-face interviews with 100 farmers were conducted during the community’s dip day. Using dipping days was key to avoiding selection bias because most farmers participate in dipping sessions on dipping days.

To maintain consistency, most questionnaires were written in a closed format, except for the introduction. In cases where the expected responses were not in-depth, an option for “others: please specify” was provided. The questions were organized in sections to collect socio-economic data and information regarding animal husbandry practices. Farm-level data were collected, such as dip tank names, local municipalities, GPS coordinates, and vegetation types. At each dip tank, five cattle owners were randomly selected to participate in the study. Interviews were conducted using the vernacular Xhosa language. Four trained enumerators, with an animal health technician associated with each dip tank, were employed to conduct the interviews. Before any data collection commenced, the farmers were informed of the purpose of the study and were guaranteed that their involvement would be voluntary and would be kept confidential.

The first section covered sociodemography, such as the farmer’s name, age, gender, level of education, and monthly income. This was followed by the characteristics of livestock production, such as the number of livestock species kept, the effects of ticks, TBD prevalence (using the vernacular, farmers were asked about diseases prevalent in their area, hence TBD prevalence), and the management of grazing areas. In addition, questions were asked regarding tick control methods, the frequency of dipping in summer and winter, management of dipping services, and whether the existing dipping compound was working efficiently or not. The second part of the questionnaire referred to alternative measures for tick challenges and knowledge of commonly observed ticks and species. The study was conducted concurrently with the field data collection of ticks, and farmers were requested to visually identify the species of cattle ticks that they encountered based on illustrations of common tick species found in the study areas. The last section of the questionnaire focused on climate change’s impact on livestock production.

### Statistical analysis

All survey and observational data were entered into Microsoft Excel^®^ 2016 (Microsoft, Washington, USA) and analyzed using STATISTICA software (://statistica.software.informer.com/10.0/). Descriptive statistics, including frequencies, means, and standard errors, were used to summarize socio-demographic data, livestock numbers, and TBD prevalence.

To examine associations between categorical variables such as gender, education level, livestock ownership, tick infestation rates, and disease prevalence, Pearson’s Chi-square (χ^2^) test was applied. This test determined whether significant relationships existed between different vegetation types and factors such as tick infestation severity and the frequency of disease occurrence.

For comparisons between multiple vegetation types, Kruskal-Wallis non-parametric analysis of variance (ANOVA) was employed. This test assessed differences in livestock numbers, TBD prevalence, and tick control methods across the ACB, AMG, BT, and GFT regions. *Post-hoc* multiple comparison tests were performed where significant differences were detected.

To compare numerical variables such as cattle ownership per farmer, tick infestation levels, and acaricide effectiveness ratings, Mann-Whitney U-tests were used where applicable, as the data did not meet the assumptions for parametric testing. The statistical significance level was set at α = 0.05, and all tests were conducted at a 95% confidence interval.

## RESULTS

### Socio-demographic profile

**Figure 2:**
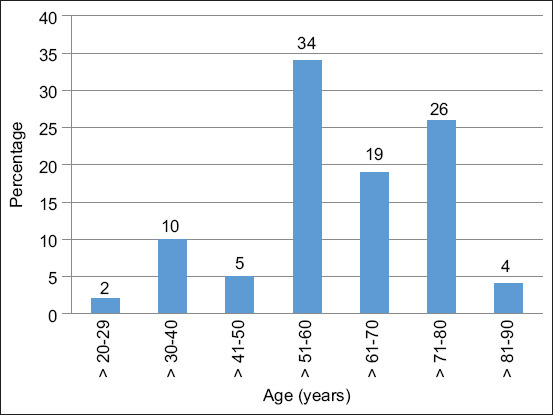
Age profile of stock owners who responded to a questionnaire survey at communal dip tanks in the Eastern Cape Province of South Africa.

A total of 100 individual questionnaires were completed (25 in each of the four vegetation types). All respondents were directly involved in livestock practices, with 43% being members of local farmer associations. The average age of all farmers was 60.49 years, with an average age of 61.47 years for women and 60.32 years for men. The respondents were mostly males (85%) compared with females (15%), and there were no significant differences between the age groups of farmers in the four vegetation types (p = 0.195). Most stock owners were older than 50 years (83%), while only 12% were younger than 40 years (Figure 2).

Less than 10% of respondents completed secondary school, 65% had some form of education up to the primary level, and 26% had no school education (Table 1). The major source of income for the cattle farmers was government pension grants (70%), with 30% of respondents solely dependent on income generated through cattle farming (Table 1). The monthly incomes of most of the participating farmers were <R 2000 (approximately 105.97 USD) (Table 1).

**Table 1:** Educational and socioeconomic status of respondents from four vegetation types in the Eastern Cape Province of South Africa.

Vegetation types	Level of education (%)				Income range per month		
	
	None	Primary	Secondary	Tertiary	<R500	R500-R2,000	>R2,000
Coastal Belt	7	18	0	0	10	15	0
Grassland	5	16	4	0	4	20	1
Thornveld	5	18	2	0	10	14	1
Thicket	9	13	3	0	6	18	1
Total	26	65	9	0	30	67	3

### Livestock records

All respondents owned cattle that grazed on communal lands throughout the year. Most (98%) of the cattle farmers also owned more than 10 goats, while 49% owned sheep, and 27% owned other livestock species (e.g., horses, donkeys, and pigs). There were no significant differences in the mean number of cattle (p = 0.596) and goats (p = 0.524), but there was a significant difference in the mean number of sheep (p < 0.001) between the different vegetation types (Table 2). The mean number of cattle per farmer ranged between 12.8 ± 1.17 and 15.6 ± 1.35 for the different vegetation types (Table 2).

**Table 2:** Mean numbers of cattle, goats, and sheep (±SE) per vegetation type in the Eastern Cape Province of South Africa.

Variable	Coastal belt	Grassland	Thornveld	Thicket
Cattle	15.6^a^ ± 1.35	12.8^a^ ± 1.17	14.4^a^ ± 1.27	14.3^a^ ± 1.47
Goats	16.1^a^ ± 1.95	12.4^a^ ± 1.88	14.2^a^ ± 1.16	15.4^a^ ± 2.00
Sheep	0.0^a^	28.9^b^ ± 4.78	3.9^a^ ± 2.70	40.2^b^ ± 4.20

^a,b^Means within the same row with different superscripts are significantly different (p < 0.001). Coastal Belt=Albany coastal belt, Grassland=Amathole montane grassland, Thornveld=Bhisho thornveld, Thicket=Great fish thicket, SE=Standard erres

### Knowledge of ticks and related TBD

A large portion of the respondents stated that they inspected their cattle for ticks on a monthly basis (75%) and confirmed that adult animals were more affected by ticks than calves (77%, χ^2^ = 15.98, p < 0.001). With the use of booklets with local tick species presented to farmers during the dipping day, as the study was conducted concurrently with the field tick collection day. However, the udder (86%) and scrotum (73%) regions of cattle are reported to be mostly affected by ticks. Almost all respondents (>90%) claimed to be able to distinguish between different tick species, with the South African bont tick *A. hebraeum* (44%) and blue tick *Rhipicephalus* (*Boophilus*) spp. (36%) reported as the most common tick species. Other tick species that were less frequently reported included the brown ear tick, *Rhipicephalus appendiculatus* (9%), bont-legged ticks, *Hyalomma* spp. (8%) and red-legged tick, and *R. e. evertsi* (3%) across the vegetation types surveyed. All the respondents identified redwater as the most common TBD, followed by gallsickness (90%) and heartwater (43%). (Table 3). Redwater and gallsickness (Anaplasmosis) were the most common TBDs within and between vegetation types, whereas heartwater (also known as cowdriosis) was less commonly reported and absent in AMG (Table 3). Farmers in BT and GFT reported higher cattle mortality than farmers in AMG and the ACB. However, no significant differences in tick-related deaths were observed among the four veld types (p = 0.081). Farmers were allowed to make differences in TBD symptoms in their region using their vernacular language. Regarding redwater, the respondents mentioned that symptoms included red urine, lack of appetite, and difficult walking. In contrast, aggressiveness and lack of appetite were mentioned for gallsickness. Furthermore, they explained that animals walk in circles, and some foam in their mouths and nostrils may appear to indicate heartwater diseases.

**Table 3:** Proportion of cattle deaths because of TBDs in four different vegetation types in the Eastern Cape Province, South Africa.

Vegetation types	Gallsickness (%)	Heartwater (%)	Redwater (%)
Coastal belt	88	68	100
Grassland	92	0	100
Thornveld	84	32	100
Thicket	96	72	100
Mean	90	43	100

Coastal Belt=Albany coastal belt, Grassland=Amathole montane grassland, Thornveld=Bhisho thornveld, Thicket=Great fish thicket, TBDs=Tick-borne diseases

### Perceptions about tick control practices

**Figure 3:**
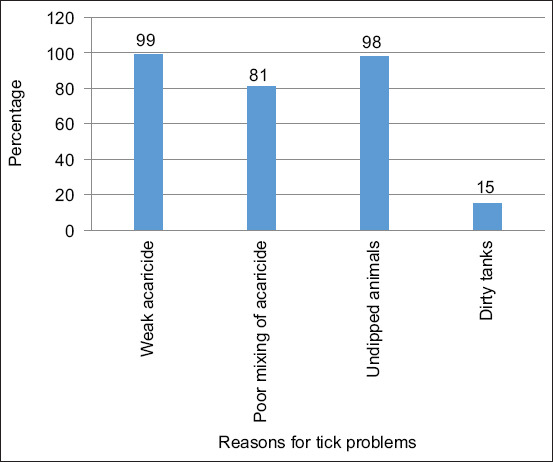
Farmer-reported constraints on effective tick control in the Eastern Cape Province, South Africa.

Forty percentages of the respondents reported that farming with adaptive breeds, such as Nguni and Brahman cattle, reduces ticks and TBDs. There was no significant difference in this respect between the respondents for the different vegetation types (χ^2^ = 7.00, p = 0.07). Farmers that treat their animals with acaricides prefer plunge dipping (90%) over hand spraying (10%). Animals were dipped once a week and more often in summer (68%) than winter (50%). Almost 60% of the respondents were not aware of restrictions on animal movement between the districts and that such actions may complicate tick control due to the introduction of resistant tick species. More than 70% of the respondents claimed that the current acaricides that were used in dip tanks were not effective in killing ticks and listed ineffective acaricides, undipped animals, and poor mixing of acaricides as the most important constraints of effective tick control practices (Figure 3).

Most farmers supplement the government dip using alternative tick control practices. Products listed as effective against ticks included old car engine oil (54%), and the household disinfectant, Jeyes fluid (35%). The use of engine oil varied between vegetation types (χ^2^ = 9.82, p < 0.05) with 76%, 56%, 52%, and 32% of the respondents from Grassland, Coastal Belt, Thornveld, and Thicket, respectively. Likewise, veld management differed significantly (χ^2^ = 11.75, p < 0.05) in terms of veld burning, with 28%, 56%, 64%, and 28% of the respondents from the ACB, AMG, BT, and GFT, respectively, while no significance difference (χ^2^ = 1.14, p = 0.77) were recorded for rotational grazing between the vegetation types.

### Farmers’ perceptions of climate change

More than half (66%) of respondents were concerned about climate change and were aware of changes in weather patterns over the past 20 years. In support of this, nearly all respondents (99%) were convinced that increases in tick counts were associated with climate change. Cattle farmers from both BT and GFT have reported that climate change has resulted in either long wet or long dry seasons (40% and 60%, respectively). All the cattle farmers in the different vegetation types observed a delay in the onset of the rainy seasons, shorter rain seasons, and warmer winters.

## DISCUSSION

From the study, it is evident that cattle farmers were mostly males as opposed to females. This can be attributed to the fact that males traditionally regard themselves as stock owners and the physical challenges associated with managing cattle in general and at dip tanks. This pattern is consistent with previous studies in the eastern and central regions of the ECP [20–22] and studies conducted in Nigeria [23] and Tanzania [24]. Most respondents in the study were older than 50 years, and 25% were between 71 and 80 years old (Figure 2). This is a concern regarding the sustainability of livestock farming in communal areas and supports previous studies that observed poor participation by youth in agricultural activities in other rural areas [20, 25]. This pattern may be associated with the high cost of living because youth and adults should provide income for themselves and their families. However, they are forced to leave rural villages (where there is little or no work) and move to cities and towns in search of employment. In contrast, the elderly receive a monthly pension that is supplemented by cattle farming and/or subsistence crop farming. Another concerning factor is the limited scholarly training that most respondents have: more than half of the respondents (65%) had primary education, while a quarter (26%) had no school training. These results are in contrast with those of Malusi *et al*. [21], who reported that in Zimbabwe, most (>90%) of the household members had formal training up to the secondary level. Lack of basic education may influence a farmer’s decisions regarding accurate dosages and other components of animal health programs, as in most cases, the farmer is responsible for mixing the dipping compounds at the dip tanks.

Communal cattle farmers were aware that several tick species infest cattle. The predominant tick species reported by farmers were *A. hebraeum*, *Rhipicephalus* (*Boophilus*) spp., and *R. appendiculatus*, whereas *Hyalomma* spp. and *R. evertsi evertsi* were less commonly observed. The tick species reported in the present study appear to be widely distributed in the ECP [26, 27] and were also recorded in previous studies in the ECP [28–30] and the eastern part of the Free State Province [11]. All reported tick species are associated with TBDs and tick-induced toxicosis [31]. Given the high prevalence of blue ticks (*Rhipicephalus* [*Boophilus*] spp.), it is not surprising that redwater and gallsickness were commonly recorded. Interestingly, heartwater incidence was predominantly recorded in communities within lower-lying vegetation types (ACB and GFT <200 m). In contrast, there is a lower incidence of heartwater (in BT) and absence (in Grassland) in communities at higher elevations (>450 m). It is possible that the vector (*A. hebraeum*) was less abundant at higher elevations, which is consistent with previous records suggesting that *A. hebraeum* is dependent on a combination of trees, shrubs, and grass for cover; as such, the tick is absent from grass-dominated vegetation found at higher elevations [30]. The two vegetation types at lower elevations (Coastal Belt and Thicket) are close to the coast where environmental conditions, such as temperature, may be more stable. Therefore, the preference of *A. hebraeum* for lower elevations may be linked to vegetation and climatic factors. In support of this, *A. hebraeum* ticks were also absent in cattle examined at two high-altitude communities (>1,400 m) in the ECP [29]. In this study, mortality associated with TBDs was higher in GFT and BT than in other vegetation types. These vegetation types have more structured vegetation cover, climatic conditions that are less the same and likely host abundance that is similar to other types. With the exception of *A. hebraeum*, there is less tick abundance along the coast, and this is also the condition in more inland grassland areas [29, 30].

The most widely used method for the control of ticks is the direct application of acaricides to host animals, and in communal farming areas of the ECP, farmers are subsidized by the government for dipping in to control ticks in their cattle. In the present study, farmers perceived that the subsidized compound Triatix 500 TR^®^ (Amitraz, Afrivet, South Africa) was weak and, therefore, ineffective in killing ticks. These findings concur with Makwarela *et al*. [8], who reported that respondents complained about weak dip wash in the northeastern ECP region. Similar findings were also recorded in the eastern Free State Province, where 80% of the farmers experienced high challenges and tick-related problems with their livestock despite using commercial acaricides [32]. There may be several reasons for poor acaricide efficacy; for instance, the community member (dip attendant) responsible for mixing the acaricide may unintentionally mix the wrong concentration due to a lack of basic education. Another possible reason is that community members may intentionally mix a weaker concentration to extend the availability of the compound. All of these factors contribute to poor acaricide efficacy and resistance in tick populations, especially in one-host ticks, *R*. (*Boophilus*) *microplus* and *R*. (*Boophilus*) *decoloratus*. Of the two blue ticks that occur in South Africa, *R*. (*Boophilus*) *microplus* displays rapid adaptation to new environments [33] and resistance to acaricide [33, 34]. From the field data collected at the same localities where dip-washing was reported to be weak and ineffective, *R*. (*Boophilus*) *microplus* was the most frequently collected species. Irrespective of the reason, frequent exposure of ticks to inadequate concentrations of acaricides enables the development of acaricide resistance, a major global problem [34–36].

Almost all respondents were aware of changes in the climate and reported an increase in cold summers and warm winters. Respondents also stated that tick abundance increased over time and attributed it to climate change. Although milder, more favorable climatic conditions can cause increased fecundity and survival of ticks [3], it is possible that the combined effect of acaricide resistance and climate change may result in elevated numbers of ticks. Similar studies in Limpopo Province, South Africa, have reported long-term changes in climatic conditions, namely rainfall and temperature [37, 38]. These climatic changes are expected to primarily affect communal rangeland farmers due to a lack of resources and management technologies [39]. Although studies have recorded a relationship between tick abundance and changes in climate, it should be noted that human activities and other factors can play a role in the distribution of ticks and the diseases they transmit [40–43].

## CONCLUSION

This study provides a comprehensive assessment of communal cattle farmers’ knowledge, perceptions, and practices regarding TBDs in the ECP of South Africa. The findings highlight the significant burden of TBDs, with redwater (100%), gallsickness (90%), and heartwater (43%) being the most frequently reported diseases. The study also underscores the role of vegetation types in shaping tick distribution, with heartwater being absent in AMG. Farmers identified acaricide resistance, poor dipping infrastructure, and uncontrolled cattle movement as major challenges in tick control, leading to the adoption of alternative, often ineffective, methods such as used motor oil and household disinfectants.

The study employed structured face-to-face interviews across diverse vegetation types, ensuring a representative understanding of communal farmers’ experiences and challenges. A combination of descriptive statistics, Chi-square tests, and Kruskal-Wallis ANOVA provided a thorough examination of factors influencing tick prevalence and control practices. The study contributes valuable insights into communal farming systems, which are often overlooked despite their economic and food security importance in rural South Africa. The findings can guide policymakers in designing targeted interventions to improve tick control measures, extension services, and acaricide resistance management.

Despite its strengths, the study has some limitations. The reliance on farmers’ perceptions and recall-based responses may introduce reporting bias. The study captures a cross-sectional snapshot rather than long-term seasonal variations in tick infestations and disease prevalence. The absence of molecular diagnostics limits the ability to validate farmers’ disease identification, and the study’s geographical focus on the ECP may restrict the generalizability of findings to other regions with different climatic and socio-economic conditions.

Future research should include multi-seasonal monitoring to assess the impact of climate variability on tick distribution and TBD prevalence. Integrating molecular and serological diagnostics can enhance the accuracy of TBD prevalence estimates and validate farmers’ observations. Further investigation into acaricide resistance and alternative control methods, such as vaccine development and biological control, is necessary. Given the aging farmer population, strategies to engage younger generations and incorporate digital tools, such as mobile-based tick surveillance, should be explored. In addition, assessing the effectiveness of government-supported tick control programs and proposing sustainable improvements in communal livestock management can provide valuable policy guidance.

This study provides critical insights into the challenges of tick management in communal cattle farming. Addressing the identified gaps through research-driven interventions, farmer education, and sustainable tick control strategies will be vital for improving cattle health and productivity in South Africa’s communal farming systems.

## AUTHORS’ CONTRIBUTIONS

NN and SM: Planned and designed the study. NN: Conducted the study and collected the samples. NN and SM: Sample analysis and drafted, edited, and revised the manuscript. All authors have read and approved the final manuscript.
